# Prevalence and associations of antibiotic resistance and biofilm formation with molecular determinants in clinical *Acinetobacter baumannii* isolates

**DOI:** 10.1038/s41598-026-64464-1

**Published:** 2026-08-10

**Authors:** Ayat M. Abu-Resha, Hazem H. Saleh, Mohamed I. Abou-Dobara, Ahmed K. El-Sayed

**Affiliations:** 1 Botany and Microbiology Department, Faculty of Science, Damietta University, PO 34517, New Damietta City, Egypt; 2 Urology and Nephrology Center, Mansoura University, Mansoura, Egypt

**Keywords:** *A. baumannii*, XDR/MDR, Carbapenemase genes, Multiplex PCR, Biofilm formation, Diseases, Microbiology, Molecular biology

## Abstract

*Acinetobacter baumannii* is a notorious opportunistic pathogen commonly associated with healthcare-associated infections and multidrug resistance. Its survival in hospital environments, biofilm formation, and iron acquisition systems complicate treatment. Understanding virulence factors and resistance mechanisms is crucial for developing effective control strategies. This study investigates genetic determinants of virulence and antibiotic resistance in selected MDR/XDR clinical *A. baumannii* isolates and explores their relationship with phenotypic traits. The selected MDR/XDR clinical *A. baumannii* isolates were identified using standard microbiological methods. Antimicrobial susceptibility followed CLSI guidelines. Biofilm formation was assessed using the tissue culture plate method. Molecular confirmation and detection of the selected genes were performed by multiplex PCR. In this study, 150 non-duplicate MDR/XDR clinical *A. baumannii* isolates were selectively collected from routine laboratory records based on their resistance profiles. Among the selected isolates, (84%) were XDR and (16%) were MDR. *bla*_OXA-51_ and *adeA* gene were present in all selected MDR/XDR isolates (100%). Among MBL genes, *bla*_IMP_, *bla*_NDM_, and *bla*_VIM_ were detected in 82%, 64%, and 52% of the selected studied isolates, respectively, with each isolate carrying at least one or more of these genes, while *bla*_KPC_ was absent. Biofilm formation was observed in 96% of the selected MDR/XDR studied isolates, including 36% strong, 44% moderate, and 16% weak producers. Virulence genes *bas*D, *sur*A1, and *bfm*R were present in all selected MDR/XDR isolates, while *csu*E, *omp*A, and *bap* were detected in 94%, 80%, and 46%, respectively. The high detection rates of metallo-β-lactamases, biofilm formation, and iron acquisition genes among the selected MDR/XDR clinical isolates, highlight the importance of continuous surveillance and infection control measures. Notably, a significant association was observed between biofilm formation and antimicrobial susceptibility profile for most tested antibiotics. Additionally, the antimicrobial susceptibility profile of the selected MDR/XDR *A. baumannii* showed statistically significant and non-significant associations with both antibiotic resistance genes and biofilm genes.

**Supplementary Information:**

The online version contains supplementary material available at 10.1038/s41598-026-64464-1.

## Introduction

*Acinetobacter baumannii* is an alarming pathogen that poses a serious global health threat due to its ability to withstand most antibiotics. The World Health Organization (WHO) classifies it as one of the most dangerous ESKAPE pathogens^1,2^, and infections caused by it are associated with high morbidity and mortality. As a result, both the WHO and the Centers for Disease Control and Prevention (CDC) have identified *A. baumannii* as a high-priority pathogen in antimicrobial resistance research, highlighting the urgent need for new therapeutic strategies and deeper scientific investigation^3^.

This pathogen is an opportunistic agent commonly associated with severe infections that are often difficult to treat due to limited therapeutic options. It is frequently associated with healthcare-associated infections, as it can colonize and persist on medical devices and hospital surfaces such as ventilators, humidifiers, dialysis machines, and washbasins^4^. This persistence contributes to its involvement in hospital-acquired infections, including ventilator-associated pneumonia (VAP), bloodstream infections, urinary tract infections, wound and skin infections, endocarditis, osteomyelitis, septicemia, and meningitis and chronic kidney disease (CKD)^5^.

Initially, *A. baumannii* was sensitive to most antibiotics until the early 1970s. However, throughout the 1980s and 1990s^6^, it rapidly acquired resistance to multiple antibiotic classes, creating a significant burden on global healthcare systems. Carbapenems, such as meropenem and imipenem, were once the most effective treatments, but the emergence of carbapenem-resistant *A. baumannii* (CRAb) has greatly reduced their efficacy. As a result, treatment has become increasingly difficult and often relies on last-resort antibiotics such as colistin or tigecycline, guided by susceptibility testing^7^.

The growing challenge in treatment is largely driven by the diverse resistance mechanisms of *A. baumannii,* which are broadly classified into enzymatic and non-enzymatic mechanisms. Enzymatic mechanisms include class A β-lactamases (KPC, GES, SHV, TEM, CTX-M, and PER) that hydrolyze a wide range of β-lactams^8^, class B metallo-β-lactamases (IMP, VIM, NDM, SIM) that require zinc ions and confer high-level carbapenem resistance and most other β-lactams^9^, class C β-lactamases, also referred to as cephalosporinases, allow *A. baumannii* to hydrolyze third-generation cephalosporins, contributing to β-lactam resistance^10^. Additionally, class D β-lactamases, including the intrinsic OXA-51 and acquired variants such as OXA-23-like, OXA-58-like, OXA-24/40-like, OXA-235-like, and OXA-143-like, play a major role in carbapenem resistance^11^. Beyond enzymatic activity, *A. baumannii* also utilizes non-enzymatic resistance mechanisms, including reduced outer membrane permeability, modifications in penicillin-binding proteins (PBPs) that lower β-lactam affinity^12^, and overexpression of multidrug efflux pumps. Among these, the AdeABC efflux pump system of the RND family is highly significant^13^. *Acinetobacter baumannii’s* capacity to form biofilms may enhance its persistence in clinical environments and contributes to treatment failure. Biofilm formation is a multifactorial process regulated by several key genes that collectively enhance bacterial adherence, colonization, and persistence on biotic and abiotic surfaces. Among these genes, the *bap* (biofilm-associated protein) gene encodes a cell-surface adhesion protein essential for biofilm architectural organization^14^. The surface antigen protein 1 (SurA1) has been shown to contribute to the pathogenicity of *A. baumannii*. It promotes bacterial adhesion and supports the early stages of biofilm formation^15^. The OmpA outer membrane protein, one of the most abundant in *A. baumannii*, acts as a major virulence factor. It plays a central role in adhesion to host epithelial cells via fibronectin binding and to abiotic surfaces, supporting colonization and biofilm establishment. Additionally, OmpA promotes immune evasion by increasing resistance to serum-mediated killing. It can also localize to host cell mitochondria, where it disrupts membrane potential and induces caspase-mediated apoptosis, contributing to tissue damage during infection^16^.

The CsuA/BABCDE chaperone–usher (Csu) pilus system is essential during the initial attachment phase of biofilm formation on abiotic surfaces, particularly medical devices. Within this system, CsuE functions as the tip adhesin required for pilus assembly and initial surface contact^17^. Regulation of the *csu* operon is mediated by the BfmRS two-component system, where BfmS serves as the sensor kinase and BfmR as the response regulator controlling expression of pilus biogenesis genes^18^. This regulatory cascade links environmental signals to surface adhesion, promoting biofilm initiation and persistence. The *basD* gene is required for the biosynthesis of the siderophore acinetobactin, which enables iron acquisition in iron-limited host environments^19^. This system enhances survival, virulence, and indirectly contributes to biofilm formation and long-term persistence^20^.

This study aimed to characterize selected MDR/XDR clinical *A. baumannii* isolates at both molecular and phenotypic levels. This would be performed by detecting and evaluating relationships between antibiotic resistance and biofilm forming genes.

## Materials and methods

### Specimen collection and processing

In this descriptive cross-sectional investigation, a total of 150 non-duplicate clinical *A. baumannii* isolates exhibiting MDR or XDR phenotypes (one per patient) were selectively obtained from different patients aged between 12 and 73 years. The isolates were selected based on their antimicrobial resistance profiles (MDR/XDR) from routine clinical laboratory records. Sampling was conducted at local clinical labs in Mansoura and Damietta governorates, Egypt, during the period from July 2024 to September 2025. Written informed consent had been obtained from patients or their legal guardians (for participants under 18 years of age) at the time of original sample collection as part of routine clinical diagnostic procedures. Clinical specimens were collected from various sources including urine, wound swabs, sputum, and bloodstream samples. The study was conducted in accordance with the declaration of Helsinki. The experimental protocol was approved by the local ethical committee (Research Ethics for Basic Sciences Committee, Damietta University, Egypt).

All selected MDR/XDR samples were cultured on conventional bacteriological media, including Brain Heart Infusion agar (Oxoid, UK) as a non-selective enrichment medium, CHROMagar *Acinetobacter* (CHROMagar, France) as a selective medium, blood agar base (Oxoid, UK) supplemented with 5% defibrinated sheep blood, MacConkey agar (Oxoid, UK) as a differential and selective medium, and cysteine electrolyte-deficient (CLED) agar (Oxoid, UK). The plates were incubated aerobically at 37 °C and examined for growth after 24 and 48 h. Pure isolates were stored in 30% glycerol at − 80 °C for further investigations. The clinical isolates were initially identified using conventional microbiological and biochemical methods^21,22^. Subsequently, species identification was confirmed using the VITEK®2 microbial identification system (bioMérieux, France) with VITEK®2 GN ID cards (bioMérieux, France), according to the manufacturer’s instructions^23^.

### The antimicrobial susceptibility testing

Antimicrobial susceptibility testing of the selected MDR/XDR isolates was performed using the VITEK®2 automated system (bioMérieux, France) with AST-GN73 susceptibility cards, designated for Gram-negative bacteria. Quality control strains were *Escherichia coli* ATCC 10536, *Pseudomonas aeruginosa* ATCC 27853, and *Klebsiella pneumoniae* ATCC 700603. All quality control results were within the acceptable ranges recommended by CLSI and were appropriate for the tested methods. Bacterial suspensions were prepared from overnight nutrient agar cultures and adjusted to a turbidity equivalent to 0.5 McFarland standard before card inoculation, following the manufacturer’s instructions. The antibiotics tested included ampicillin/sulbactam (SAM), piperacillin/tazobactam (TZP), ceftazidime (CAZ), ceftriaxone (CRO), cefepime (FEP), meropenem (MEM), gentamicin (GEN), tobramycin (TOB), ciprofloxacin (CIP), levofloxacin (LEV), and trimethoprim/sulfamethoxazole (SXT). The system measured bacterial growth across a range of antibiotic concentrations to determine the minimum inhibitory concentrations (MICs). The MIC (µg/mL) susceptibility breakpoints for all tested antimicrobial agents are presented in Supplementary Table S1 and were interpreted according to CLSI 2021 (M100, 31st Edition) criteria^24^, as the VITEK®2 system software was validated and calibrated to this version, which was the current interpretive standard available at the testing facility during the study period. Each isolate was classified as susceptible, intermediate, or resistant based on these criteria. For association analyses, intermediate and resistant categories were combined and considered non-susceptible.

### Metallo-ß-lactamase (MBL) production test

The production of metallo-β-lactamase (MBL) was assessed using the combined disk test on Mueller–Hinton agar (MHA) plates. In this method, imipenem and imipenem–EDTA (Ethylene diamine tetra acetic acid) disks were used, with 0.5 M EDTA serving as the chelating inhibitor, following established protocols^25^.

### Detection of biofilm formation using tissue culture plate method

Biofilm formation was evaluated using the tissue culture plate (TCP) method, a widely used quantitative in vitro assay for biofilm detection, as previously described by Christensen et al.^26^. Fresh bacterial colonies were inoculated into Tryptic Soy Broth (TSB) supplemented with 1% glucose and incubated at 37 °C for 24 h. The cultures were then diluted 1:100, and 200 µL of each bacterial suspension was transferred into sterile flat-bottom 96-well microtiter plates. Negative control wells containing sterile broth only supplemented with 1% glucose without bacterial inoculation, were included in each experiment. Following incubation at 37 °C for 24 h, non-adherent cells were removed by washing with phosphate-buffered saline (PBS). Adherent biofilms were fixed with 99% methanol for 15 min and stained with 0.1% crystal violet. Excess stain was removed by washing with deionized water, and the bound dye was resolubilized using 160 µL of 33% glacial acetic acid. Optical density (OD) was measured at 570 nm using a microplate ELISA reader (Model 680, Bio-Rad, UK). All experiments were performed in triplicate. Mean OD_570_ values obtained from replicate wells were used for biofilm classification. The optical density cut-off value (mean ODc: 0.11) was calculated as the mean OD_570_ of the negative control wells plus three standard deviations. Biofilm production was classified as follows: strong biofilm producer (OD_570_ > 4 × ODc), moderate biofilm producer (2 × ODc < OD_570_ ≤ 4 × ODc), weak biofilm producer (ODc < OD_570_ ≤ 2 × ODc), and non-biofilm producer (OD_570_ ≤ ODc). The observed OD ranges for each biofilm category in the selected MDR/XDR isolates are provided in Supplementary Table S2.

### Genomic DNA extraction

Genomic DNA was extracted using the phenol–chloroform method as described by^27^. Then the DNA quality and integrity was assessed by electrophoresis on 1% agarose gel. All procedures were performed under aseptic conditions to minimize contamination.

### Multiplex PCR

In this study, two distinct multiplex PCR assays were performed to characterize the virulence and antibiotic-resistance determinants of the selected MDR/XDR clinical *A. baumannii* isolates. PCR amplification was performed using a thermal cycler (Mastercycler Nexus Thermal Cycler, Eppendorf, Germany). Multiplex PCR was carried out to detect the virulence genes (*omp*A*, bap, csu*E*, sur*A1*, bfm*R*,* and *bas*D) under the following cycling conditions: initial denaturation at 94 °C for 5 min; 30 cycles of denaturation at 94 °C for 30 s, annealing at 55 °C for 30 s, and extension at 72 °C for 1 min; followed by a final extension at 72 °C for 5 min. To evaluate the presence of class A β-lactamase (*bla*_KPC_), carbapenemase genes MBLs (*bla*_IMP_*, bla*_VIM_*,* and *bla*_NDM_), class D β-lactamase (*bla*_OXA-51_): a species-specific molecular confirmation marker, and *ade*A gene, multiplex PCR was performed under the following cycling conditions: initial denaturation at 94 °C for 5 min; 30 cycles of denaturation at 94 °C for 30 s, annealing at 54 °C for 30 s, and extension at 72 °C for 1min; followed by a final extension at 72 °C for 5 min. All PCR reactions were carried out using 2X PCR master mix (Bioline, UK) and all primers used in this study were purchased from macrogen (Seoul, South Korea) and listed in Table 1.

**Table 1:** The primers used for the amplification of antibiotic-resistance and virulence genes.

Type of genes	Function	Primer code	Primer Sequence (5′ → 3′)	PCR product size (bp)	Annealing Tm. (°C)	Refs.
Antibiotic-resistance genes	Class A β-lactamase	KPC-F	CGTCTAGTTCTGCTGTCTTG	798	54	^28^
		KPC-R	CTTGTCATCCTTGTTAGGG			
	Class B or metallo β-lactamase (MBLs)	NDM-F	GGTTTGGCGATCTGGTTTTC	621	54	^28^
		NDM-R	CGGAATGGCTCATCACGATC			
		VIM-F	GATGGTGTTTGGTCGCATA	390	54	^28^
		VIM-R	CGAATGCGCAGCACCAG			
		IMP-F	GGAATAGAGTGGCTTAATTCTC	232	54	^28^
		IMP-R	GGTTTAACAAAACAACCACC			
	Class D β-lactamase	OXA 51-F	TAATGCTTTGATCGGCCTTG	353	54	^29^
		OXA 51-R	TGGATTGCACTTCATCTTGG			
	Efflux pumps	AdeA-F	GCCACCACCGGCTAAAGTCA	654	54	^30^
		AdeA-R	GGTTGCCCGTGGCTATTGGT			
Virulence genes	Biofilm formation	CsuE-F	ACCAATGCTCAGACCGGAG	751	55	^30^
		CsuE-R	CTTGTACCGTGACCGTATCTTG			
		OmpA-F	CGCTTCTGCTGGTGCTGAAT	531	55	^31^
		OmpA-R	CGTGCAGTAGCGTTAGGGTA			
		Bap-F	ATGCCTGAGATACAAATTAT	1449	55	^32^
		Bap-R	GTCAATCGTAAAGGTAACG			
		SurA1-F	CAATTGGTAGCTGGCGATCA	241	55	^33^
		SurA1-R	TTAGGCGGGACTCAGCTTTT			
	Biofilm regulatory system	BfmR-F	GGATCTTGTGGTCTTGGATGTC	384	55	^34^
		BfmR-R	GATAAAATACGGCCAGCGTTTG			
	Siderophore production	BasD-F	CTCTTGCATGGCAACACCAC	868	55	^35^
		BasD-R	CCAACGAGACCGCTTATGGT			

PCR products were separated on 3% agarose gel prepared with 5μg/mL ethidium bromide (Thermo Fisher Scientific, USA) in TAE buffer. Electrophoresis was performed at 100 V for 45 min, and DNA bands were visualized under UV illumination. A 100 bp DNA ladder (GDSBio, China) was used as a molecular size marker.

### Statistical analysis

Statistical analysis was performed using the Statistical Package for the Social Sciences (SPSS) version 25. Associations between variables were analyzed using the Chi-square test or Fisher’s exact test when appropriate. Fisher’s exact test was applied when the expected frequency in any cell of the contingency table was small. A *p* value ≤ 0.05 was considered statistically significant.

## Results

### Demographic characteristics of the study population

**Fig. 1:**
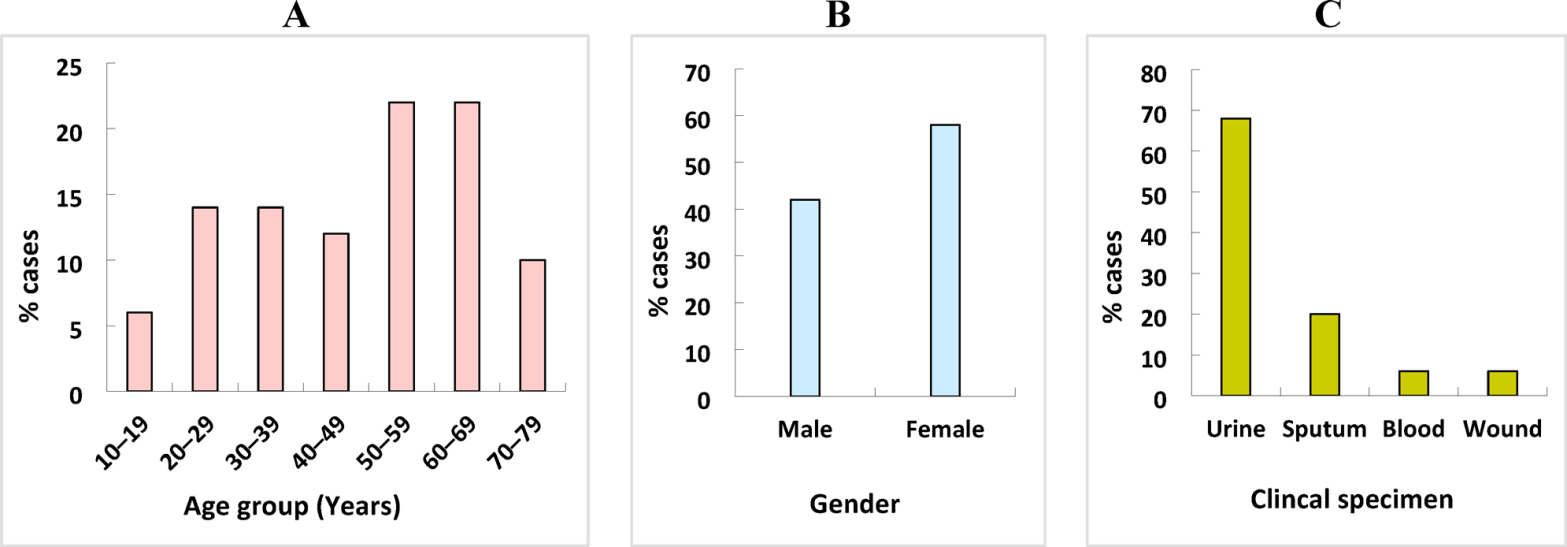
Demographic characteristics of the study population. (**A**) Age group percentage, (**B**) Gender percentage, (**C**) Clinical specimens’ percentage.

In the present study, a total of 150 non-duplicate MDR/XDR clinical *A. baumannii* isolates were selectively recovered from clinical specimens obtained from patients aged between 12 and 75 years. The age distribution of the studied patients is illustrated in (Fig. 1A). Patients were categorized into seven age groups. The highest frequencies were observed among patients aged 50–59 years and 60–69 years, with 33 patients in each group. This was followed by the 20–29 and 30–39 age groups, each comprising 21 patients. Eighteen patients belonged to the 40–49 age group, while 15 patients were aged 70–79 years. The lowest number of patients was recorded in the 10–19 age group (n = 9). A higher proportion of the selected MDR/XDR isolates was obtained from female patients (n = 87, 58%) compared to male patients (n = 63, 42%), as illustrated in (Fig. 1B). The distribution of the selected MDR/XDR clinical specimens is shown in (Fig. 1C). The majority of the selected MDR/XDR isolates were obtained from urine specimens (n = 102, 68%), followed by sputum samples (n = 30, 20%), while bloodstream and wound specimens represented the least common sources of isolation, with 9 isolates each (6%).

### Antimicrobial susceptibility test

Multidrug-resistant (MDR) and extensively drug-resistant (XDR) *A. baumannii* isolates were classified based on antimicrobial category-level nonsusceptibility according to the international standard definitions proposed by Magiorakos et al.^36^. MDR was defined as acquired non-susceptibility to at least one agent in three or more antimicrobial categories, whereas XDR was defined as non-susceptibility to at least one agent in all but two or fewer antimicrobial categories (i.e. bacterial isolates remain susceptible to only one or two categories).

**Fig. 2:**
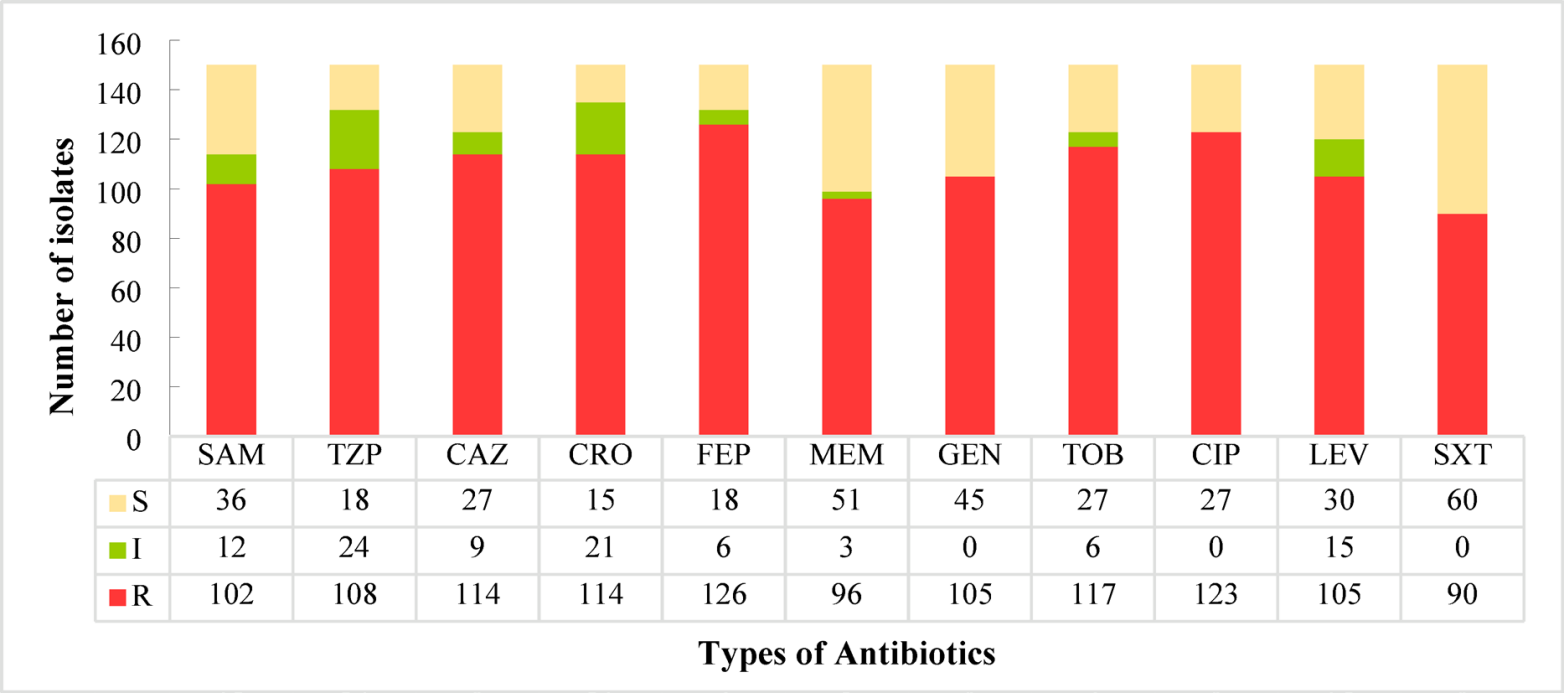
Antimicrobial susceptibility testing of 150 selected MDR/XDR clinical *A. baumannii* isolates, SAM (ampicillin/sulbactam), TZP (piperacillin/tazobactam), CAZ (ceftazidime), CRO (ceftriaxone), FEP (cefepime), MEM (meropenem), GEN (gentamicin), TOB (tobramycin), CIP (ciprofloxacin), LEV (levofloxacin), SXT (trimethoprim/sulfamethoxazole), S (sensitive), I (intermediate), R (resistant).

In the present study, among the selected MDR/XDR clinical *A. baumannii* isolates, 126 (84%) were classified as XDR, and 24 (16%) were MDR. The antimicrobial susceptibility testing revealed widespread resistance across multiple antibiotic classes. Among penicillin and β-lactam/β-lactamase inhibitor combinations, high resistance rates were observed for piperacillin–tazobactam (72%) and ampicillin–sulbactam (68%). Within the cephalosporin group, cefepime (84%), ceftazidime (76%), and ceftriaxone (76%) showed substantial resistance. For carbapenems, meropenem resistance was observed in 64% of isolates. Among aminoglycosides, resistance affected 78% of isolates for tobramycin and 70% for gentamicin. Fluoroquinolones also showed significant resistance, with ciprofloxacin at 82% and levofloxacin at 70%. Finally, trimethoprim–sulfamethoxazole displayed the lowest resistance among all tested agents, affecting 60% of isolates. These findings are summarized in Table 2 and illustrated in Fig. 2, which shows the antimicrobial susceptibility profile of the selected MDR/XDR clinical *A. baumannii* isolates.

**Table 2:** Antimicrobial susceptibility profile of 150 selected MDR/XDR clinical *A. baumannii* isolates.

Antibiotic class	Antibiotic (abbreviation)	(S) n (%)	(I) n (%)	(R) n (%)
Penicillins + β-lactamase inhibitor	Ampicillin–sulbactam (SAM)	36 (24%)	12 (8%)	102 (68%)
	Piperacillin–tazobactam (TZP)	18 (12%)	24 (16%)	108 (72%)
Cephalosporins	Ceftazidime (CAZ)	27 (18%)	9 (6%)	114 (76%)
	Ceftriaxone (CRO)	15 (10%)	21 (14%)	114 (76%)
	Cefepime (FEP)	18 (12%)	6 (4%)	126 (84%)
Carbapenems	Meropenem (MEM)	51 (34%)	3 (2%)	96 (64%)
Aminoglycosides	Gentamicin (GEN)	45 (30%)	0 (0%)	105 (70%)
	Tobramycin (TOB)	27 (18%)	6 (4%)	117 (78%)
Fluoroquinolones	Ciprofloxacin (CIP)	27 (18%)	0 (0%)	123 (82%)
	Levofloxacin (LEV)	30 (20%)	15 (10%)	105 (70%)
Others (Folate pathway inhibitor**)**	Trimethoprim–sulfamethoxazole (SXT)	60 (40%)	0 (0%)	90 (60%)

*S* sensitive, *I* intermediate, *R* resistant, *n* number of isolates.

### Metallo-ß-lactamase (MBL) test

**Fig. 3:**
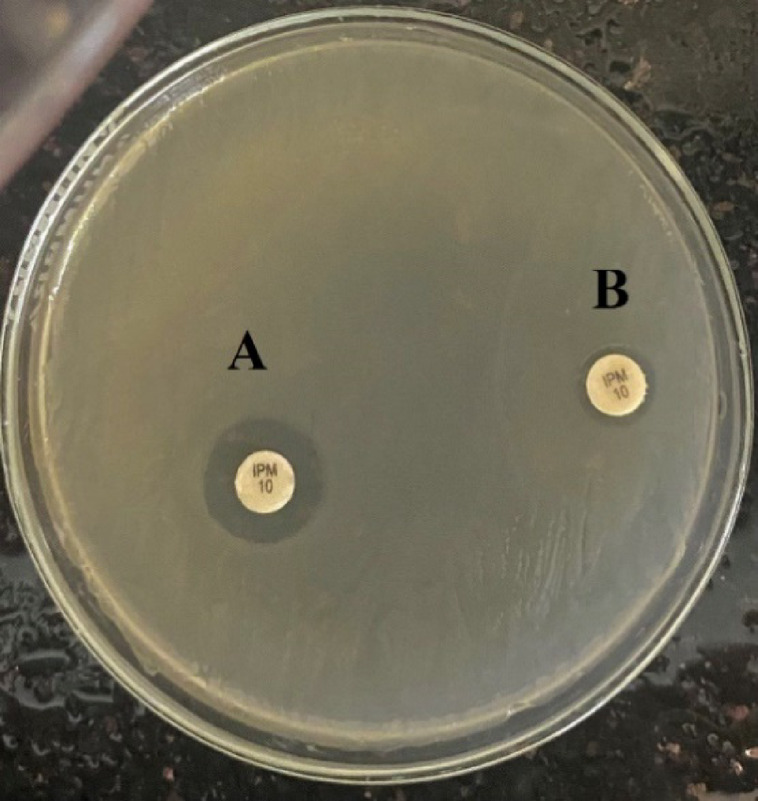
Representative *A. baumannii* isolate for Metallo-ß-lactamase test. (**A**) EDTA-IMP disc showing a clear inhibition zone ≥ 7 mm, indicating a positive result for Metallo-β-lactamase production. (**B**) IMP disc alone, reflecting resistance of the isolate to imipenem.

All 150 selected MDR/XDR clinical *A. baumannii* isolates were MBL-positive by the combined disk test, showing an increase of ≥ 7 mm in inhibition zones with imipenem-EDTA disks (Fig. 3). The phenotypic and genotypic comparison of MBL detection among the studied *Acinetobacter baumannii* isolates is presented in Supplementary Table S3.

### Molecular detection of antibiotic resistance genes

**Fig. 4:**
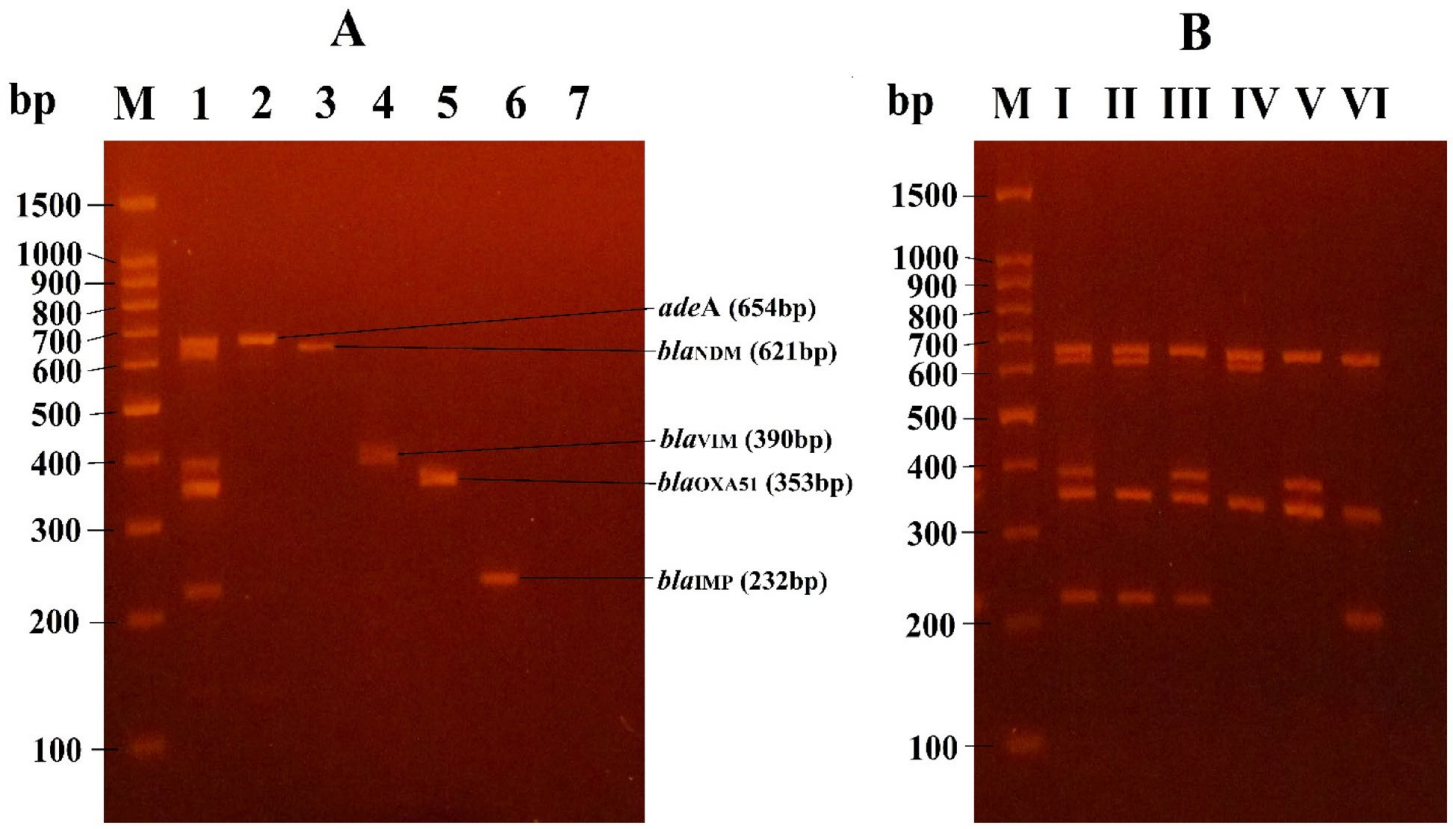
PCR products of the amplified antibiotic resistance genes on agarose gel electrophoresis. (**A**) Optimization of the PCR using previously characterized *A. baumannii* isolate, Lane 1: multiplex PCR for all antibiotic resistance genes. Lanes 2–6: uniplex PCR for *ade*A, *bla*_NDM_, *bla*_VIM_, *bla*_OXA-51_ and *bla*_IMP_, respectively. Lane 7: negative control (**B**) The multiplex PCR for the selected MDR/XDR clinical *A. baumannii* isolates showing different six profiles (see Table 3) for the antibiotic resistance gene among the selected MDR/XDR clinical *A. baumannii* isolates. M (100 bp DNA marker).

**Fig. 5:**
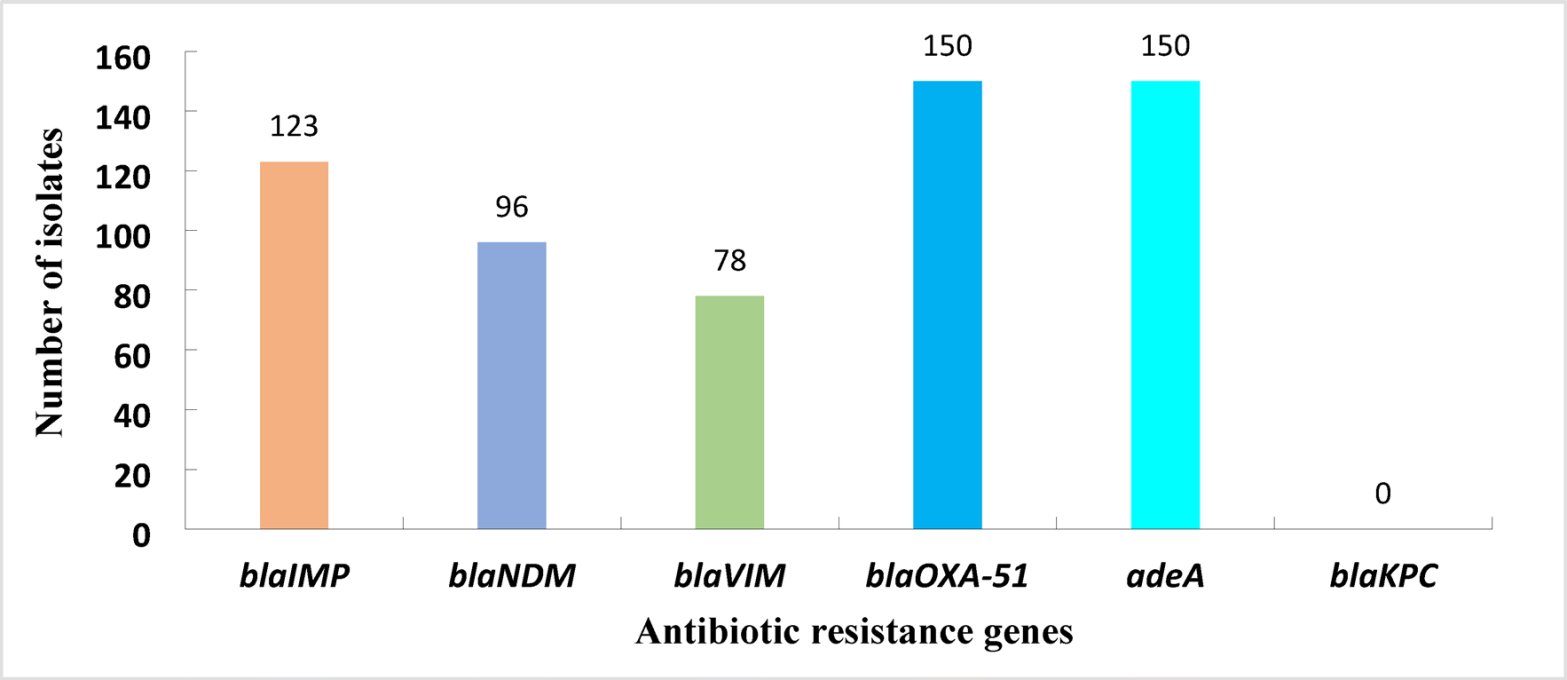
Frequency of antibiotic resistance genes among the selected MDR/XDR clinical *A. baumannii* isolates.

Previously identified and characterized *Acinetobacter baumannii* isolates harboring the targeted resistance genes (unpublished data), were obtained from the culture collection of the Microbiology Laboratory, Botany and Microbiology Department, Faculty of Science, Damietta University. These isolates were used as positive controls for optimization of multiplex PCR conditions and determination of the expected amplicon sizes for each gene (Fig. 4A). No-template controls (nuclease-free water instead of DNA) to detect contamination in reagents, consumables, or the laboratory environment were included in each PCR run to ensure assay specificity and exclude contamination. The multiplex PCR analysis of antibiotic resistance genes revealed six distinct profiles among the selected MDR/XDR studied isolates (Fig. 4B). Each profile was defined according to the observed amplification pattern of the investigated genes as summarized in Table 3. Subsequently, PCR screening was analyzed to determine the distribution of individual antibiotic resistance genes among the 150 selected MDR/XDR clinical *A. baumannii* isolates. The intrinsic class D β-lactamase gene *bla*_OXA-51_ was detected in all selected MDR/XDR isolates (150/150, 100%). Similarly, the *ade*A gene was identified in all studied isolates (150/150, 100%). Among MBL genes, *bla*_IMP_ was detected in 123 isolates (82.0%), followed by *bla*_NDM_ in 96 isolates (64.0%) and *bla*_VIM_ in 78 isolates (52.0%). In contrast, the class A carbapenemase gene *bla*_KPC_ was not detected in any of the selected MDR/XDR studied isolates (Fig. 5).

**Table 3:** Resistance gene profiles and their frequency resulting in multiplex PCR for antibiotic resistance genes of 150 selected MDR/XDR clinical *A. baumannii* isolates.

Pattern	Gene profile	No. of genes	No. of isolates	(%)
P I	*ade*A—*bla*_NDM_—*bla*_VIM_—*bla*_OXA-51_—*bla*_IMP_	5	48	32
P II	*ade*A—*bla*_NDM_—*bla*_OXA-51_—*bla*_IMP_	4	27	18
P III	*ade*A—*bla*_VIM_—*bla*_OXA-51_—*bla*_IMP_	4	24	16
P IV	*ade*A—*bla*_NDM_—*bla*_OXA-51_	3	21	14
P V	*ade*A—*bla*_VIM_—*bla*_OXA-51_	3	6	4
P VI	*ade*A—*bla*_OXA-51_—*bla*_IMP_	3	24	16

### Biofilm formation strength

**Fig. 6:**
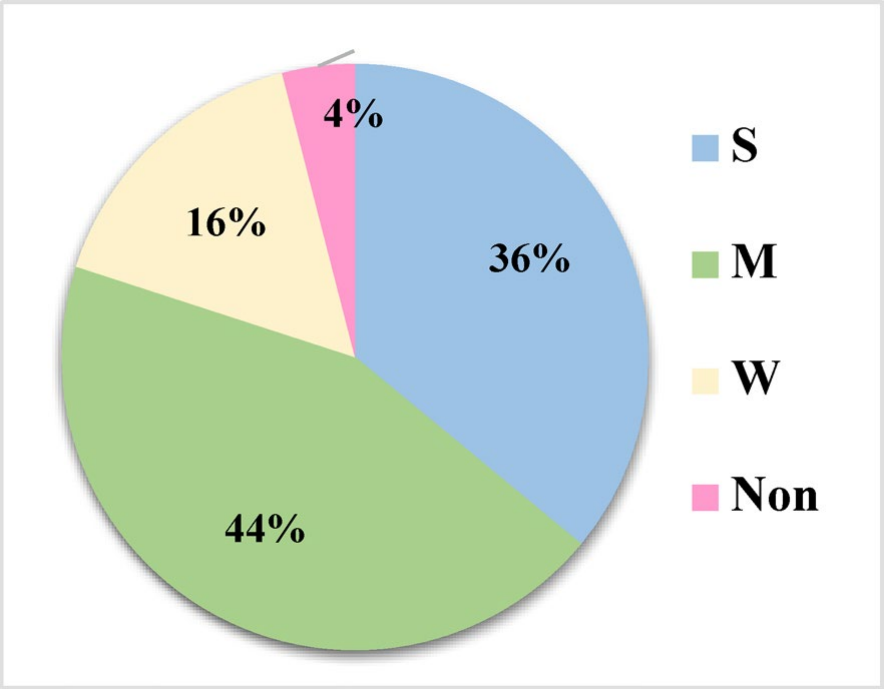
Biofilm formation strength of the 150 selected MDR/XDR *A. baumannii* isolates, S (strong), M (moderate), W (weak).

Out of the selected MDR/XDR clinical *A. baumannii* isolates, 144 (96.0%) demonstrated the ability to form biofilm, whereas only six isolates (4.0%) were classified as non-biofilm producers. Among the biofilm-forming selected MDR/XDR isolates, 54 (36.0%) were identified as strong, 66 (44.0%) as moderate and 24 (16.0%) as weak biofilm producers (Fig. 6), indicating that moderate biofilm formation was the most prevalent phenotype.

### Molecular detection of virulence-associated genes

**Fig. 7:**
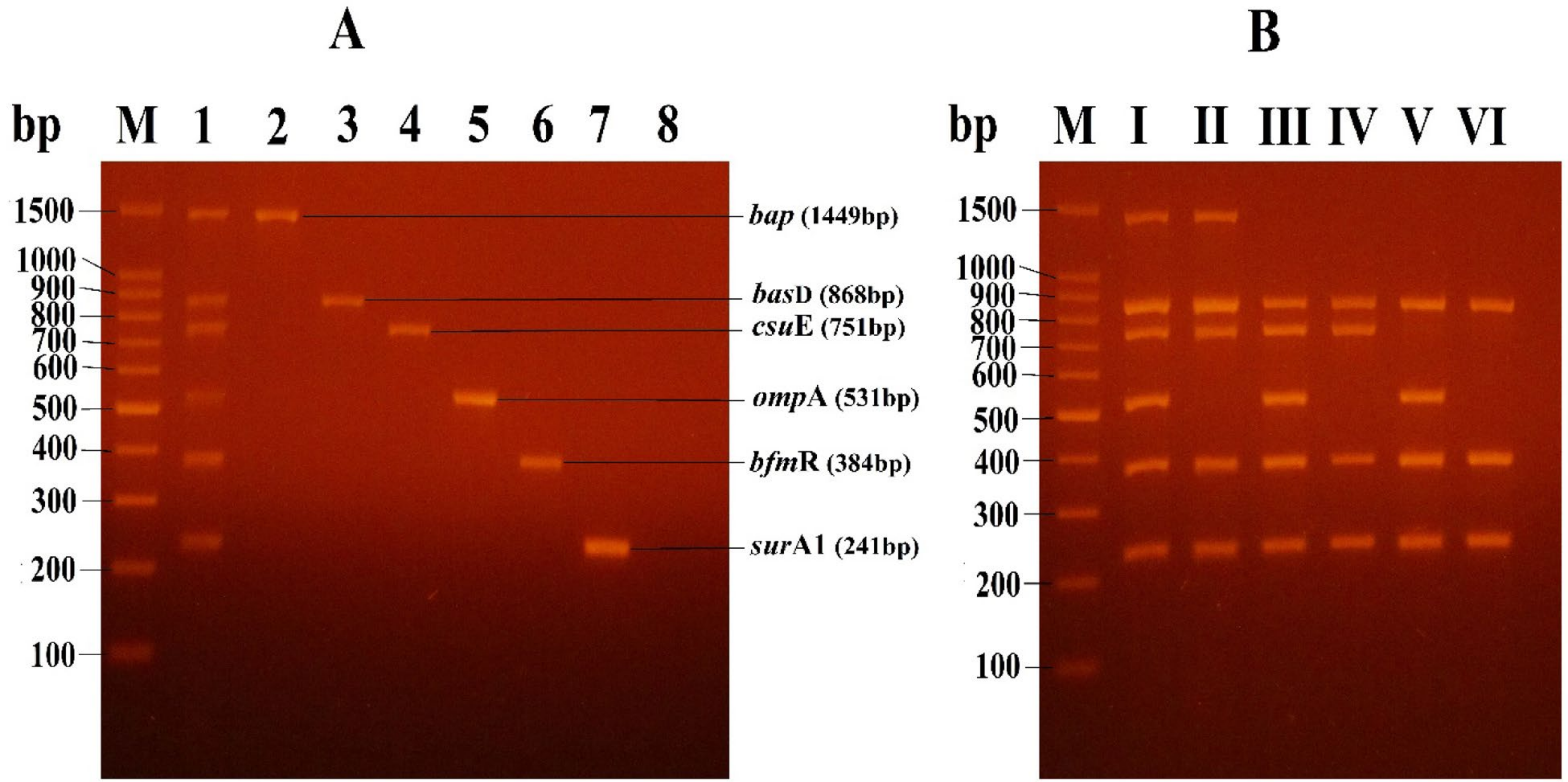
PCR products of the amplified virulence associated genes on agarose gel electrophoresis. (**A**) Optimization of the PCR using previously characterized *A. baumannii* isolate, Lane 1: multiplex PCR for all virulence associated genes. Lanes 2–7: uniplex PCR for *bap*, *bas*D, *csu*E, *omp*A, *bfm*R and *sur*A1, respectively. lane 8: negative control (**B**) The multiplex PCR for the selected MDR/XDR *A. baumannii* isolates showing different six profiles, (see Table 4) for the virulence associated genes among the selected MDR/XDR clinical *A. baumannii* isolates.

**Fig. 8:**
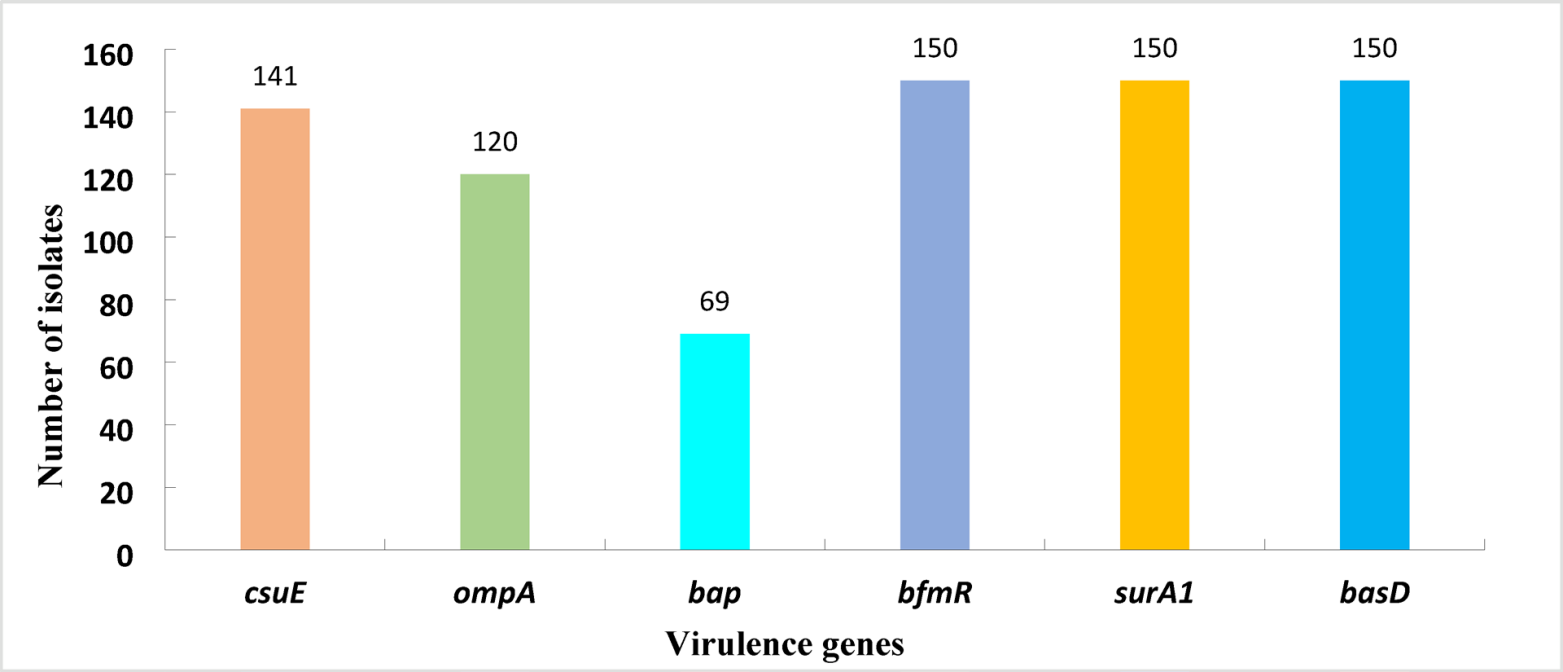
Frequency of virulence genes among the selected MDR/XDR *A. baumannii* isolates.

Multiplex PCR was performed to investigate the presence and distribution of virulence-associated genes among the selected MDR/XDR clinical *A. baumannii* isolates. The previously characterized *A. baumannii* isolate carrying the targeted virulence genes was used to optimize the multiplex PCR conditions and to determine the expected amplicon size for each gene (Fig. 7A). No-template controls (nuclease-free water instead of DNA) to detect contamination in reagents, consumables, or the laboratory environment were included in each PCR run to ensure assay specificity and exclude contamination. The multiplex PCR analysis of virulence genes revealed six distinct profiles among the selected MDR/XDR studied isolates (Fig. 7B). Each profile was defined according to the observed amplification pattern of the investigated genes as summarized in Table 4. Molecular analysis revealed a high detection rate of biofilm-related determinants among the selected studied isolates, with most of the studied isolates harboring multiple biofilm-associated genes. Specifically, *bfm*R and *sur*A1 were detected in all selected isolates (150/150, 100%). The *csu*E gene was identified in 141 isolates (94%), while *omp*A was detected in 120 isolates (80%). The *bap* gene showed the lowest frequency among the studied biofilm-associated genes, being present in 69 isolates (46%). Additionally, the siderophore-associated gene *bas*D was detected in all selected isolates (150/150, 100%) (Fig. 8).

**Table 4:** Virulence associated gene profiles identified by multiplex PCR in the selected MDR/XDR *A. baumannii* isolate.

Profile	Gene profile	No. of genes	No. of isolates	%
P I	*bap*—*bas*D—*csu*E—*omp*A—*bfm*R—*sur*A1	6	63	42
P II	*bap*—*bas*D—*csu*E—*bfm*R—*sur*A1	5	6	4
P III	*bas*D—*csu*E—*omp*A—*bfm*R—*sur*A1	5	51	34
P IV	*bas*D—*csu*E—*bfm*R—*sur*A1	4	21	14
P V	*bas*D—*omp*A—*bfm*R—*sur*A1	4	6	4
P VI	*bas*D—*bfm*R—*sur*A1	3	3	2

### Correlation between antimicrobial resistance pattern (MDR & XDR) and biofilm formation

A statistically significant association was observed between biofilm formation and antimicrobial resistance pattern. However, this finding should be interpreted with caution due to the small number of non-biofilm-producing isolates (n = 6), all of which were MDR, and the preselection of isolates based on MDR/XDR phenotypes, as shown in Table 5.

**Table 5:** Correlation between antimicrobial resistance pattern (MDR & XDR) and biofilm formation.

XDR-MDR (No. of isolates)	Biofilm formation (No. of isolates)		*P* value
	Biofilm forming (144)	Non-Biofilm forming (6)	
XDR (126)	126	0	< 0.001
MDR (24)	18	6	
Total (150)	144	6	

(*P* ≤ 0.05) is considered statistically significant.

### Correlation between biofilm formation and antimicrobial susceptibility profile

As shown in Table 6, a significant association was observed between biofilm formation and antimicrobial susceptibility profile for most tested antibiotics. Significant associations were detected for ampicillin/sulbactam (SAM), ceftazidime (CAZ), cefepime (FEP), meropenem (MEM), gentamicin (GEN), tobramycin (TOB), ciprofloxacin (CIP), levofloxacin (LEV), and trimethoprim/sulfamethoxazole (SXT), while no significant association was observed for piperacillin/tazobactam (TZP) and ceftriaxone (CRO).

**Table 6:** Correlation between biofilm formation and antimicrobial susceptibility profile.

Type of antibiotic	Antibiotic susceptibility profile	Biofilm formation (No. of isolates)		*P* value
		Biofilm forming (144)	Non-biofilm forming (6)	
SAM	S (36)	30	6	< 0.001
	NS (114)	114	0	
TZP	S (18)	18	0	1.000
	NS (132)	126	6	
CAZ	S (27)	21	6	< 0.001
	NS (123)	123	0	
CRO	S (15)	15	0	1.000
	NS (135)	129	6	
FEP	S (18)	12	6	< 0.001
	NS (132)	132	0	
MEM	S (51)	45	6	0.001
	NS (99)	99	0	
GEN	S (45)	39	6	0.001
	NS (105)	105	0	
TOB	S (27)	21	6	< 0.001
	NS (123)	123	0	
CIP	S (27)	21	6	< 0.001
	NS (123)	123	0	
LEV	S (30)	24	6	< 0.001
	NS (120)	120	0	
SXT	S (60)	54	6	0.004
	NS (90)	90	0	

(*P* ≤ 0.05) is considered statistically significant.

*S* susceptible, *Ns* non-susceptible.

### Correlation between antibiotic resistance genes and antimicrobial susceptibility profile

As shown in Table 7, the association between antimicrobial susceptibility profile and β-lactamase genes was evaluated. For *bla*_IMP_, significant associations were observed with ceftazidime (CAZ), ceftriaxone (CRO), meropenem (MEM), and ciprofloxacin (CIP) (*P* < 0.05), while no significant associations were detected for the remaining antibiotics. For *bla*_NDM_, significant associations were observed with ampicillin/sulbactam (SAM), ceftazidime (CAZ), and meropenem (MEM) (*p* ≤ 0.05), whereas no significant associations were observed for the other antibiotics. Regarding *bla*_VIM_, significant associations were observed with cefepime (FEP), tobramycin (TOB), and trimethoprim/sulfamethoxazole (SXT) (*p* < 0.05), while no significant associations were detected for the remaining antibiotics. Overall, gene distribution showed variable associations across different antimicrobial classes.

**Table 7:** Correlation between antibiotic resistance genes and antimicrobial susceptibility profile.

Types of antibiotics	Antibiotic susceptibility profile	Antibiotic resistance genes (No. of isolates)								*p* value
		*bla*_IMP_ (123)		*p* value	*bla*_NDM_ (96)		*p* value	*bla*_VIM_ (78)		
		Yes	No		Yes	No		Yes	No	
SAM	S (36)	27	9	0.220	18	18	0.049	21	15	0.446
	NS (114)	96	18		78	36		57	57	
TZP	S (18)	12	6	0.098	12	6	1.000	9	9	1.000
	NS (132)	111	21		84	48		69	63	
CAZ	S (27)	15	12	< 0.001	12	15	0.026	12	15	0.404
	NS (123)	108	15		84	39		66	57	
CRO	S (15)	9	6	0.031	12	3	0.258	9	6	0.593
	NS (135)	114	21		84	51		69	66	
FEP	S (18)	12	6	0.098	9	9	0.200	3	15	0.002
	NS (132)	111	21		87	45		75	57	
MEM	S (51)	36	15	0.013	27	24	0.050	27	24	1.000
	NS (99)	87	12		69	30		51	48	
GEN	S (45)	36	9	0.651	30	15	0.713	21	24	0.476
	NS (105)	87	18		66	39		57	48	
TOB	S (27)	21	6	0.581	18	9	0.827	6	21	0.001
	NS (123)	102	21		78	45		72	51	
CIP	S (27)	15	12	< 0.001	15	12	0.377	12	15	0.404
	NS (123)	108	15		81	42		66	57	
LEV	S (30)	21	9	0.066	15	15	0.090	15	15	0.840
	NS (120)	102	18		81	39		63	57	
SXT	S (60)	45	15	0.084	39	21	0.864	39	21	0.012
	NS (90)	78	12		57	33		39	51	

(*p* ≤ 0.05) is considered statistically significant.

*S* susceptible, *Ns* non-susceptible.

### Correlation between antibiotic resistance genes and biofilm formation

A significant association was observed between biofilm formation and *bla*_VIM_ gene, while no significant associations were detected for *bla*_IMP_ or *bla*_NDM_, as shown in Table 8.

**Table 8:** Correlation between antibiotic resistance genes and biofilm formation.

Biofilm formation (No. of isolates)	Antibiotic resistance genes (No. of isolates)								*p* value
	*bla*_IMP_ (123)		*p* value	*bla*_NDM_ (96)		*p* value	*bla*_VIM_ (78)		
	Yes	No		Yes	No		Yes	No	
Biofilm forming (144) Non-Biofilm forming (6)	120	24	0.072	93	51	0.668	78	66	0.011
	3	3		3	3		0	6	

(*p* ≤ 0.05) is considered statistically significant.

### Correlation between virulence genes and biofilm formation

A significant association was observed between biofilm formation and *csu*E gene, while no significant associations were detected for *omp*A or *bap* genes, as shown in Table 9.

**Table 9:** Correlation between virulence genes and biofilm formation.

Biofilm formation (No. of isolates)	Virulence genes (No. of isolates)								
	*csu*E (141)		*p* value	*omp*A (120)		*p* value	*Bap* (69)		*p* value
	Yes	No		Yes	No		Yes	No	
Biofilm forming (144) Non-Biofilm forming (6)	138	6	0.003	117	27	0.095	66	78	1.000
	3	3		3	3		3	3	

(*p* ≤ 0.05) is considered statistically significant.

### Correlation between virulence genes and antimicrobial susceptibility profile

The association between antibiotic susceptibility phenotypes and virulence genes (*csu*E, *omp*A, and *bap*) was evaluated. For *csu*E, significant correlations were observed with ampicillin/sulbactam (SAM), ceftazidime (CAZ), meropenem (MEM), ciprofloxacin (CIP), and levofloxacin (LEV), while no significant correlations were observed with the remaining antibiotics. For *omp*A, significant correlations were identified with ceftazidime (CAZ), gentamicin (GEN), and trimethoprim/sulfamethoxazole (SXT), while no significant correlations were observed with the remaining antibiotics. Regarding *bap*, significant correlations were observed with tobramycin (TOB) and trimethoprim/sulfamethoxazole (SXT), while no significant correlations were observed with the remaining antibiotics as shown in Table 10.

**Table 10:** Correlation between virulence genes and antimicrobial susceptibility profile.

Types of antibiotics	Antibiotic Susceptibility profile	Virulence genes (No. of isolates)								
		*csu*E (141)		*p* value	*omp*A (120)		P value	*Bap* (69)		*p* value
		Yes	No		Yes	No		Yes	No	
SAM	S (36)	27	9	< 0.001	27	9	0.473	18	18	0.702
	NS (114)	114	0		93	21		51	63	
TZP	S (18)	15	3	0.077	12	6	0.204	6	12	0.317
	NS (132)	126	6		108	24		63	69	
CAZ	S (27)	21	6	0.001	15	12	0.001	9	18	0.201
	NS (123)	120	3		105	18		60	63	
CRO	S (15)	15	0	0.599	9	6	0.080	3	12	0.053
	NS (135)	126	9		111	24		66	69	
FEP	S (18)	15	3	0.077	12	6	0.204	12	6	0.078
	NS (132)	126	6		108	24		57	75	
MEM	S (51)	42	9	< 0.001	36	15	0.052	24	27	0.864
	NS (99)	99	0		84	15		45	54	
GEN	S (45)	42	3	1.000	30	15	0.013	24	21	0.285
	NS (105)	99	6		90	15		45	60	
TOB	S (27)	24	3	0.206	21	6	0.792	18	9	0.020
	NS (123)	117	6		99	24		51	72	
CIP	S (27)	21	6	0.001	18	9	0.066	12	15	1.000
	NS (123)	120	3		102	21		57	66	
LEV	S (30)	24	6	0.002	24	6	1.000	15	15	0.685
	NS (120)	117	3		96	24		54	66	
SXT	S (60)	54	6	0.157	42	18	0.021	21	39	0.031
	NS (90)	87	3		78	12		48	42	

(*p* ≤ 0.05) is considered statistically significant.

*S* susceptible, *Ns* non-susceptible.

### Co-occurrence between virulence genes and antibiotic resistance genes

The association between β-lactamase genes and virulence genes was evaluated. For *bla*_IMP_, significant associations were observed with *csu*E and *omp*A, while no significant association was detected with *bap*. For *bla*_NDM_, no significant associations were observed with any of the virulence genes. Regarding *bla*_VIM_, a significant association was observed with *bap*, whereas no significant associations were detected with *csu*E or *omp*A. as illustrated in Table 11.

**Table 11:** Co-occurrence between virulence genes and antibiotic resistance genes.

Virulence genes (No. of isolates)		Antibiotic resistance genes (No. of isolates)								
		*bla*_IMP_ (123)		*p* value	*bla*_NDM_ (96)		*p* value	*bla*_VIM_ (78)		*p* value
		+	−		+	−		+	−	
*bap* (69)	+	60	9	0.201	42	27	0.498	24	45	< 0.001
	*−*	63	18		54	27		54	27	
*csu*E (141)	+	120	21	0.001	93	48	0.071	72	69	0.498
	−	3	6		3	6		6	3	
*omp*A (120)	+	105	15	0.001	72	48	0.055	63	57	0.840
	−	18	12		24	6		15	15	

(*p* ≤ 0.05) is considered statistically significant. + (positive), − (negative).

### Association between sample source and virulence genes

The association between sample source and biofilm genes (*csu*E, *omp*A, and *bap*) was evaluated. No significant association was observed for *csu*E and *omp*A with sample source, while *bap* showed a significant association, with higher distribution in urine and respiratory isolates compared to blood and wound samples.as showed in Table 12.

**Table 12:** Association between sample source and virulence genes.

Sample source (No. of isolates)	virulence genes (No. of isolates)								
	*csu*E (141)		*p* value	*omp*A (120)		*p* value	*Bap* (69)		*p* value
	Yes	No		Yes	No		Yes	No	
Urine (102)	93	9	0.362	78	24	0.103	48	54	0.014
Sputum (30)	30	0		27	3		15	15	
Blood (9)	9	0		9	0		0	9	
Wound (9)	9	0		6	3		6	3	

(*p* ≤ 0.05) is considered statistically significant.

### Association between sample source and antibiotic resistance genes

The association between sample source and β-lactamase genes was evaluated. No significant associations were observed for *bla*_IMP_ and *bla*_VIM_, while *bla*_NDM_ showed a significant association with sample source as showed in Table 13.

**Table 13:** Association between sample source and antibiotic resistance genes.

Sample source (No. of isolates)	Antibiotic resistance genes (No. of isolates)								
	*bla*_IMP_ (123)		*p* value	*bla*_NDM_ (96)		*p* value	*bla*_VIM_ (78)		*p* value
	Yes	No		Yes	No		Yes	No	
Urine (102)	84	18	0.325	57	45	0.006	54	48	0.571
Sputum (30)	24	6		24	6		15	15	
Blood (9)	9	0		6	3		6	3	
Wound (9)	6	3		9	0		3	6	

(*p* ≤ 0.05) is considered statistically significant.

## Discussion

Over the past decades, *A. baumannii* has gained increasing attention as a critical priority pathogen in healthcare settings worldwide. The global rise of multidrug-resistant *A. baumannii* represents a significant clinical challenge. Its combined capacity for antimicrobial resistance acquisition and biofilm formation might play a pivotal role in therapeutic failure and persistent hospital transmission. In the present study, a higher proportion of the selected MDR/XDR *A. baumannii* isolates was observed among females compared to males. Similar findings regarding the predominance of female patients have been reported in Egypt, Jordan, and India^37–39^. Notably, urine samples were the most frequent source of the selected MDR/XDR isolates in this study, consistent with previous reports^38,40^. However, since detailed clinical data were not available to distinguish between true infection and colonization, particularly for urine and sputum samples, these isolates are referred to as clinical isolates obtained from various specimen types rather than as causative agents of confirmed infections. Among the selected isolates, XDR isolates were more frequent than MDR isolates, consistent with reports from several Egyptian healthcare settings^41^. Carbapenem resistance remains a major clinical concern, as these agents are among the most effective therapeutic options against *A. baumannii*. Meropenem resistance rates have been documented in previous studies, ranging from approximately 52–98% depending on the geographic region and study population^42–44^. In addition to carbapenem resistance, high resistance was observed for several antibiotic classes in our study. Higher resistance rates, up to 100%, have been reported for certain antibiotics in some studies, including piperacillin/tazobactam, cefepime, ceftazidime, ceftriaxone, gentamicin, ciprofloxacin, and levofloxacin^39,45,46^, ampicillin-sulbactam resistance in other study reached 71.7%^47^, tobramycin resistance in other study reached 78%^45^, and trimethoprim-sulfamethoxazole from 53.3 to 98%^45–47^. These variations in resistance patterns likely reflect differences in antibiotic usage, geographic location, infection control practices, and patient populations.

Regarding metallo-β-lactamase (MBL) production, all selected MDR/XDR *A. baumannii* isolates were positive by the combined disc test, and PCR confirmed that each isolate carried at least one MBL gene, with some harboring multiple genes. This widespread distribution may reflect clonal dissemination or horizontal gene transfer and is consistent with previous reports of high MBLdetection^39^. It is important to note that EDTA-based phenotypic methods have limitations, including possible false-positive results due to non-MBL mechanisms, and should therefore be interpreted alongside molecular findings.

Molecular screening demonstrated widespread distribution of resistance-associated genes among the selected MDR/XDR *A. baumannii* isolates. The intrinsic *bla*_OXA-51_ gene was detected in all selected isolates, consistent with previous studies^39,48^, which further supports its role as a reliable molecular marker for species identification within the *A. calcoaceticus–baumannii* complex, as it is intrinsic to *A. baumannii* and rarely found in other members of the complex. Similarly, the *ade*A gene was detected in all selected isolates, in agreement with several reports^49,50^, although lower frequencies have also been described^37^. A recent global systematic review identified *bla*_IMP_, *bla*_NDM_, and *bla*_VIM_ as the most commonly detected MBL genes among *A. baumannii* worldwide^51^. This study revealed that *bla*_IMP_ was the predominant gene followed by *bla*_NDM_ and *bla*_VIM_ consistent with Sharma et al.^52^. In contrast, *bla*_KPC_ was not detected, in agreement with Mahmood et al.^53^, although it has been reported in other regions in all isolates^44^. These findings reflect the geographical variability of carbapenemase genes among the selected MDR/XDR clinical *A. baumannii* isolates.

Biofilm production is an important virulence and survival mechanism in *A. baumannii*, which might contribute to antimicrobial tolerance^54^. In the present study, most of the selected MDR/XDR clinical isolates were biofilm producers, with moderate biofilm formation being the predominant phenotype. Similar findings have been reported in previous studies^55,56^, although lower rates were described elsewhere^57^.

Multiplex PCR analysis revealed that all selected MDR/XDR clinical *A. baumannii* isolates harbored the *bas*D gene, consistent with previous studies reporting similarly high detection rates^45,58^, although lower frequencies have also been described^59^. In addition, *sur*A1 and *bfm*R genes were detected in all selected MDR/XDR clinical isolates, supporting their strong conservation among the studied clinical *A. baumannii* isolates and aligning with some previous reports^59,60^. The *csu*E gene showed a higher detection rate than *omp*A, while *bap* exhibited the lowest frequency, consistent with previous studies^61,62^. High detection rates of *csu*E have also been widely reported^56,61,63^. In agreement with Zeighami et al., the *omp*A gene was detected in most of the selected studied isolates^64^, although lower frequencies were described elsewhere^47,59^. The *bap* gene showed the lowest frequency among the other studied genes, consistent with several studies^47,64,65^, while higher rates have also been reported in all isolates^45,63^. These findings reflect the variability in genes distribution among the selected MDR/XDR clinical *A. baumannii* isolates from different clinical and geographic settings. Interestingly, some of the selected MDR/XDR clinical *A. baumannii* isolates lacking some of the investigated biofilm–associated genes were still able to produce biofilm. This might be attributed to the presence of other biofilm-related genes that were not included in the current study, such as *pga*ABCD operon and *aba*I. Conversely, some of the selected isolates harboring one or more biofilm–associated genes didn’t exhibit biofilm formation, indicating that gene presence does not demonstrate overexpression or functional activity, and biofilm formation is a complex and multifactorial process^66^.

A significant association between biofilm formation and the selected MDR/XDR status was observed in this selected subset, consistent with some previous studies^67,68^. However, this finding should be interpreted cautiously, as all six non-biofilm producers were MDR, while all XDR isolates produced biofilm. The small non-biofilm group, group imbalance, and preselection of resistant isolates limit this correlation. Notably, a non-significant correlation was reported by Da Silva et al.^69^, highlighting the variability of such associations across different study populations. Additionally, biofilm formation showed a significant association with antimicrobial non-susceptibility to most tested antibiotics in this exploratory analysis, possibly reflecting biofilm-associated antibiotic tolerance, as the biofilm matrix may impair antibiotic penetration and enhance bacterial survival independently of inherited resistance mechanisms. These findings are consistent with previous studies^55,70^. However, no significant association was observed for TZP and CRO likely reflecting differences in resistance mechanisms and sample distribution. The antimicrobial susceptibility profile of *A. baumannii* was observed in relation to resistance-related genes, with both significant and non-significant associations across different antibiotics. It is important to emphasize that the association between MBL carriage and non-susceptibility to ciprofloxacin, tobramycin and trimethoprim-sulfamethoxazole is correlative, not causative, as MBL enzymes specifically degrade carbapenems and other β-lactams. Although, the underlying mechanisms were not directly examined, the observed statistical associations could be explained by possible co-carriage of resistance genes on mobile genetic elements or the dissemination of high-risk clones carrying multiple resistance determinants. Nevertheless, these interpretations require further investigation. In addition, variable statistical associations were noted between biofilm-related genes and antimicrobial susceptibility profile in the selected MDR/XDR *A. baumannii* isolates, with both significant and non-significant associations across different antibiotics, highlighting the multifactorial nature of this relationship. Biofilm formation also showed variable statistically significant and non-significant associations with both resistance and virulence genes, possibly indicating a complex and non-uniform relationship between phenotypic traits and genetic determinants. These findings are exploratory and do not imply direct causative links. The presence of genetic determinants does not always correlate with their corresponding phenotypic expression. Statistically significant and non-significant associations were also observed between virulence determinants and antibiotic resistance genes in the selected MDR/XDR *A. baumannii* isolates. Although the specific mechanisms underlying these associations were not investigated in the present study, several hypotheses could be considered. For instance, the coexistence of these determinants on mobile genetic elements (such as plasmids, integrons, or transposons) might facilitate their co-transfer. Alternatively, clonal dissemination of strains carrying combined resistance and virulence traits could contribute to their persistence in clinical settings. However, these interpretations remain speculative and require further experimental validation. Importantly, all association analyses in this study are exploratory and should be interpreted with caution due to the large number of comparisons performed.

### Limitations

The isolates were selectively collected based on MDR/XDR resistance profiles and therefore may not represent the overall distribution of clinical *A. baumannii* isolates, which may limit the generalizability and comparison with susceptible isolates. In addition, lack of clinical outcome data prevented correlation with patient outcomes, and lack of MIC distributions. Clonal relatedness was not assessed, and whole-genome sequencing was not performed. Sequencing confirmation of representative PCR amplicons was not performed. The study also included an incomplete carbapenemase gene panel, as it did not investigate major *bla*_OXA-_type carbapenemase genes that are important in carbapenem-resistant *A. baumannii*, particularly *bla*_OXA-23-_like, *bla*_OXA-24/40-_like, and *bla*_OXA-58-_like. Additionally, gene expression levels were not evaluated. Furthermore, biofilm-specific antibiotic tolerance testing was not performed.

## Conclusions

This study revealed a high frequency of antibiotic resistance, biofilm formation, and virulence-associated genes among selectively collected MDR/XDR *A. baumannii* isolates, indicating widespread distribution of resistance and virulence determinants in the studied population. The study underscores the importance of infection control strategies and further research to elucidate the mechanisms underlying resistance and biofilm formation.

## Supplementary Information

Below is the link to the electronic supplementary material.

Supplementary Material 1

Supplementary Material 2

Supplementary Material 3

Supplementary Material 4

Supplementary Material 5

## Data Availability

All data generated or analysed during this study are included in this published article.

## Abbreviations

CLSI: Clinical and Laboratory Standards Institute
WHO: The World Health Organization
CDC: Centers for Disease Control and Prevention
VAP: Ventilator-associated pneumonia
CKD: Chronic kidney disease
CRAb: Carbapenem-resistant *A. baumannii*
PBPs: Penicillin-binding proteins
RND: Resistance–nodulation–division
MICs: The minimum inhibitory concentrations
MHA: Mueller–Hinton agar
TSB: Tryptic soy broth
EDTA: Ethylene diamine tetra acetic acid
TAE: Tris–acetate–EDTA
DNA: Deoxyribonucleic acid
ESKAPE: *Enterococcus faecium, Staphylococcus aureus, Klebsiella pneumoniae, Acinetobacter baumannii, Pseudomonas aeruginosa and Enterobacter* Species
MBL: Metallo-β-lactamase
IMP: Imipenemase
KPC: *Klebsiella pneumoniae* Carbapenemase
NDM: New Delhi MBL
VIM: Verona integron-encoded MBL
OXA: Oxacillinase
PCR: Polymerase chain reaction
SurA1: Surface antigen protein 1
OmpA1: Outer membrane protein
Bap: Biofilm associated protein
MDR: Multidrug-resistant
XDR: Extensively drug-resistant
S: Susceptible
I: Intermediate
R: Resistant
μg: Microgram
