# Supplementary material for: Prevalence and associations of antibiotic resistance and biofilm formation with molecular determinants in clinical Acinetobacter baumannii isolates
Source: Sci Rep. 2026 Aug 10;16:24726. doi: 10.1038/s41598-026-64464-1 (PMC13458683; doi:10.1038/s41598-026-64464-1)
Supplement: Supplementary file 1 [file 41598_2026_64464_MOESM5_ESM.pdf]

Supplementary Table S1.

Antimicrobial susceptibility breakpoints (MIC values, µg/mL) for tested antibiotics according to CLSI 2021 guidelines.

Antimicrobial class	Antibiotic	Symbol	MIC (ug/ml)		
			S	I	R
β-lactam / β-lactamase inhibitor	Ampicillin/sulbactam	SAM	≤ 4	8-16	≥ 32
	Piperacillin/tazobactam	TZP	≤ 16	32-64	≥ 128
Cephalosporins	Ceftazidime	CAZ	≤ 8	16	≥ 32
	Ceftriaxone	CRO	≤ 8	32	≥ 64
	Cefepime	FEP	≤ 4	8	≥ 32
Carbapenems	Meropenem	MEM	≤ 4	8	≥ 16
Aminoglycosides	Gentamycin	GEN	≤ 4	8	≥ 16
	Tobramycin	TOB	≤ 4	8	≥ 16
Fluoroquinolone	Ciprofloxacin	CIP	≤ 1	2	≥ 4
	Levofloxacin	LEV	≤ 1	2	≥ 4
Folate pathway inhibitor	Trimethoprim/Sulphamethoxazole	SXT	≤ 1	2	≥ 4

Supplementary Table S2

Observed OD_{570} ranges for biofilm classification among the studied *Acinetobacter baumannii* isolates (n=150).

Criteria for Biofilm classification	Theoretical OD_{570} values	Observed OD_{570} range	N (%)
Strong biofilm producer ($OD_{570} > 4\times ODc$)	($OD_{570} > 0.44$)	(0.511 - 1.203)	54 (36%)
Moderate biofilm producer ($2\times ODc < OD_{570} \leq 4\times ODc$)	($0.22 < OD_{570} \leq 0.44$)	(0.250 - 0.392)	66 (44%)
Weak biofilm producer ($ODc < OD_{570} \leq 2\times ODc$)	($0.11 < OD_{570} \leq 0.22$)	(0.152 - 0.198)	24 (16%)
Non-biofilm producer ($OD_{570} \leq ODc$).	($OD_{570} \leq 0.11$)	(0.064 - 0.081)	6 (4%)

N (number of isolates)

Supplementary Table S3

Comparison between phenotypic MBL detection and genotypic MBL gene distribution among *Acinetobacter baumannii* isolates.

**Genotypic detection of MBL using Multiplex PCR**					**Phenotypic detection of MBL using Combined Disk Test**	
Pattern	Gene Profile	No. of genes	No. of isolates	Genotype (%)	No. of isolates	Phenotype (%)
P I	*bla*$_{NDM}$ - *bla*$_{VIM}$ - *bla*$_{IMP}$	3	48	32	48	32
P II	*bla*$_{NDM}$ - *bla*$_{IMP}$	2	27	18	27	18
P III	*bla*$_{VIM}$ - *bla*$_{IMP}$	2	24	16	24	16
P IV	*bla*$_{NDM}$	1	21	14	21	14
P V	*bla*$_{VIM}$	1	6	4	6	4
P VI	*bla*$_{IMP}$	1	24	16	24	16
